# Research Progress on the Synergistic Anti-Tumor Effect of Natural Anti-Tumor Components of Chinese Herbal Medicine Combined with Chemotherapy Drugs

**DOI:** 10.3390/ph16121734

**Published:** 2023-12-15

**Authors:** Hongrui Zhou, Mengxue Zhang, Huihui Cao, Xintong Du, Xin Zhang, Jin Wang, Xiuli Bi

**Affiliations:** 1 College of Life Science, Liaoning University, Shenyang 110036, China; 2 Key Laboratory for Chronic Diseases Molecular Mechanism Research and Nutritional Intervention of Shenyang, Shenyang 110036, China

**Keywords:** natural anti-tumor ingredients, Chinese herbal medicine, chemotherapy, combination therapy

## Abstract

The application of chemotherapy drugs in tumor treatment has a long history, but the lack of selectivity of drugs often leads to serious side effects during chemotherapy. The natural anti-tumor ingredients derived from Chinese herbal medicine are attracting increased attention due to their diverse anti-tumor effects, abundant resources, and minimal side effects. An effective anti-tumor strategy may lie in the combination of these naturally derived anti-tumor ingredients with conventional chemotherapy drugs. This approach could potentially inhibit tumor growth and the development of drug resistance in tumor cells while reducing the adverse effects of chemotherapy drugs. This review provides a comprehensive overview of the combined therapy strategies integrating natural anti-tumor components from Chinese herbal medicine with chemotherapy drugs in current research. We primarily summarize various compounds in Chinese herbal medicine exhibiting natural anti-tumor activities and the relevant mechanisms in synergistic anti-tumor combination therapy. The focus of this paper is on underlining that this integrative approach, combining natural anti-tumor components of Chinese herbal medicine with chemotherapy drugs, presents a novel cancer treatment methodology, thereby providing new insights for future oncological research.

## 1. Introduction

Cancer is a highly prevalent malignant disease, and currently chemotherapy is still the main treatment method for various cancers, with long-term proven clinical effects that can save many lives and extend the lives of many patients [1]. The traditional chemotherapy treatment method is to use certain drugs that can destroy and interfere with cell growth and survival to kill cancer cells and block their division and growth [2]. However, chemotherapy is a double-edged sword. In addition to the advantages of anti-tumor effects, chemotherapy has the disadvantages of poor selectivity and non-targeted distribution [3]. Chemotherapy drugs cannot effectively recognize normal and cancer cells, so in addition to being able to eliminate cancer cells, they can also harm cells that grow and divide normally in the body [4].

Traditional Chinese medicine is an important component of modern medicine and has also played a crucial role in anti-tumor treatment in recent years. Chinese herbal medicines with good therapeutic effects and few side effects have attracted increasing attention [5]. Traditional Chinese medicine posits that tumors are malignant formations caused by various factors such as insufficient Qi, endogenous harmful energy, the imbalance of Qi-blood, and visceral function disorders. In traditional Chinese medicine, the principle of treating tumors is dialectical treatment, which strives to restore the balance of Yin and Yang according to the individual patient conditions, strengthen the positive Qi, eliminate negative Qi, and ultimately support health while suppressing malignancy. While the Chinese herbal medicines can directly or indirectly affect tumor cells, the therapeutic efficacy of using them alone seems limited [6]. Most of the anti-tumor active ingredients in traditional Chinese medicine have the characteristics of poor solubility and significant toxic side effects, which are often limited in clinical application [7].

With the progress of modern medical technology, the progress of separation and purification technology and the in-depth study of drug action mechanism, the active ingredients of traditional Chinese medicine (such as curcumin, Andrographide, and Tanshinone IIA) have become more prominent in tumor treatment [8], and the concept of combined therapy has appeared in anti-tumor research [9]. However, combination therapy is not a simple aggregation of various treatments, but rather a comprehensive consideration of the characteristics and specific circumstances of each patient, facilitating informed and strategic therapeutic choices. While pursuing an extended survival period, attention should also be paid to improving the patients’ quality of life. Research has shown that traditional Chinese medicine can potentially increase sensitivity, enhance anti-tumor effects, and alleviate adverse reactions such as cancer-related fatigue and bone marrow suppression caused by chemotherapy drugs, thereby significantly improving the treatment outcome [10]. Consequently, the strategy of integrating chemotherapy drugs with natural anti-tumor ingredients of Chinese herbal medicine is gradually emerging as a new approach for cancer treatment.

In this review, we summarize the active ingredients of traditional Chinese medicine with anti-tumor therapy and outline the synergistic anti-tumor mechanism of natural anti-tumor components in traditional Chinese medicine combined with various chemotherapy drugs. Emphasis was placed on the potential contribution of active ingredients in traditional Chinese medicine in cancer combination therapy, as well as elucidating the relevant molecular mechanisms of their synergistic effects with chemotherapy drugs. Finally, the challenges and future prospects of combining traditional Chinese medicine active ingredients with chemotherapy drugs for anti-tumor treatment are proposed, in order to provide better strategies for optimizing tumor treatment.

## 2. Natural Anti-Tumor Ingredients Extracted from Chinese Herbal Medicine

The natural extracts of Chinese herbal medicine have been employed for anti-tumor treatment in China for thousands of years. In-depth research has led to the development of certain Chinese herbal medicines into clinically approved anticancer drugs [11]. Further investigations have revealed an abundant of natural anti-tumor agents present in Chinese herbal medicine [12]. Based on their source and mechanism of action, these natural anti-tumor components in Chinese herbal medicine are categorized into the following groups: polyphenols, terpenoids, alkaloids, and polysaccharides. The anti-tumor mechanisms vary among these natural compounds found in Chinese herbal medicines. The following summary provides reported examples of natural extracts from Chinese herbal medicines exhibiting anti-tumor effects.

### 2.1. Polyphenol Compounds

Genistein, abundantly found in leguminous plants (*Fabaceae*), was discovered to inhibit the growth of cancer cells without toxicity [13]. Li et al. [14] demonstrated in 2006 that genistein inhibits osteoclast bone resorption and prostate cancer bone metastasis through regulation of OPG/RANK/RANKL/MMP-9 signaling in a human experimental bone metastasis model. Further advancing our understanding of natural anti-cancer compounds, silymarin, a flavonoid compound extracted from the silybum marianum fruit (*Silybum marianum*), was proven by Ahmad et al. [15] in 2017 to increase the strength of reactive oxygen species and chromatin aggregation, hence exhibiting efficient anti-tumor effects. In 2021, Wang et al. [16] has summarized the role of isoliquiritigenin derived from licorice plants (*Glycyrrhiza uralensis*) in cancer. Concurrently, formononetin, an isoflavone found in Astragalus membranaceus (*Astragalus membranaceus*), Sophora flavescens (*Sophora flavescens*), Glycyrrhiza uralensis (*Glycyrrhiza uralensis*), and Pueraria lobata (*Pueraria lobata*) [17], was recognized by Wang et al. [18] for its anti-tumor potential. Formononetin could inhibit the occurrence of cervical cancer by downregulating MYC and STAT3, thereby interfering with the activation of PD-L1. Another compound, puerarin, is a natural flavonoid derived from Pueraria lobata (*Pueraria lobata*) and has high anti-tumor and anti-inflammatory properties. Chen et al. [19] in 2022 investigated the therapeutic effect of puerarin on bladder cancer and its underlying molecular mechanism. Their research confirmed puerarin’s ability to regulate the proliferation, apoptosis, and aging of T24 and EJ cells via miR-139-5p/CCNB1 and PI3K/AKT pathways. Moreover, icariin, a natural flavonoid from Epimedium plant (*Epimedium brevicornum*), has also gained attention. Fu et al. [20] confirmed in 2022 that icariin inhibits the growth of ovarian cancer tumors by targeting miR-1-3p/TNKS2/Wnt/β-catenin signaling. Beyond common flavonoids, there are many polyphenolic compounds with anti-tumor activity in Chinese herbal medicine. For example, tanshinone IIA, a natural quinone compound extracted from Salvia miltiorrhiza (*Salvia miltiorrhiza*), was found by Liu et al. [21] to inhibit the human nasopharyngeal carcinoma cell proliferation and induce apoptosis through p53-Cyclin B1/CDC2 mediated signaling. Similarly, Rhein, a natural quinone compound extracted from traditional Chinese medicine rhubarb (*Rheum officinale*), was identified by Jiang et al. [22] in 2022 to potentially induce liver cancer cell apoptosis. Furthermore, curcumin, a hydrophobic polyphenolic compound from turmeric rhizome (*Curcuma longa*), was shown by Wang et al. [23] to inhibit gastric cancer cell proliferation by inducing apoptosis and stimulating immune cells to secrete numerous cytokines. Honokiol, a polyphenol compound from traditional Chinese medicine Magnolia officinalis (*Magnolia officinalis*), was demonstrated by Hsiao et al. [24] to significantly increase reactive oxygen species in BFTC-905 tumor cells, leading to mitochondrial hyperpolarization and functional disorders, and ultimately tumor cell proliferation inhibition, as summarized in Table 1.

### 2.2. Terpenoid

Ginsenoside Rg3, a compound derived from ginseng (*Panax ginseng*), has been widely employed in oncological research. In 2009, Tang et al. [35] demonstrated this compound could exert anti-tumor effects in primary breast cancer through the inhibition of angiogenesis, cell cycle progression, and the induction of cell apoptosis. Furthermore, oridonin, a biologically active diterpenoid of lupine and laurane, is the main component of the traditional Chinese medicine oridonin (*Rabdosia rubescens*). Li et al. [36] first evaluated the anti-proliferative properties of derivatives of oridonin in 2016, showing stronger anti-tumor activity than paclitaxel.

Tripterygium wilfordii A, another natural terpenoid compound, is extracted from Tripterygium wilfordii plants (*Tripterygium wilfordii*). Cheng et al. [37] confirmed in 2016 that Triptolide A inhibits the activation of NF-κB pathway, thereby sensitizing human breast cancer cells to tumor necrosis factor-α-induced cell apoptosis. A triterpenoid compound found in Ganoderma lucidum (*Ganoderma lucidum*) has also demonstrated potential in anti-tumor drug development. Du et al. [38] explored the anti-tumor activity of Ganoderma lucidum triterpenes in 2017, establishing a foundation for the future research in this area. In addition, Wang et al. [39] reported in 2018 that oleanolic acid could inhibit the epithelial mesenchymal transition process in liver cancer cells by attenuating their infiltration and migration ability, ultimately inhibiting tumor growth. Glycyrrhetinic acid, a biologically active glycoside extracted from licorice (Glycyrrhiza uralensis) belonging to the triterpenoid class of compounds, has shown promise in cancer immunotherapy or adjuvant therapy. In 2020, Juin et al. [40] found that glycyrrhetinic acid exhibited anticancer effects on melanoma. Subsequent studies revealed that this compound can enhance anti-tumor immunity by inhibiting T regulatory cells and Myeloid-derived suppressor cells, suggesting significant potential for future oncological research, as summarized in Table 2.

### 2.3. Alkaloid Compounds

Camptothecin, a pentacyclic quinoline alkaloid and an active component in Chinese herbal medicine, is extracted from the fruit of Camptotheca acuminata (*Camptotheca acuminata*). Clinical trials have shown that camptothecin and its analogues exhibit effective anti-tumor activity. Lorence et al. [48] in 2004 confirmed that camptothecin exert anticancer activity via the inhibition of DNA topoisomerase, positioning it as a crucial natural anti-tumor product. Chelidonine, a bioactive substance extracted from Baiqucai (*Chelidonium majus*), was shown to have cancer-inhibiting properties. In 2020, Qian et al. [49] confirmed that chelidonine could inhibit NRAS signaling, leading to a series of NRAS mutations, reduced cancer cell proliferation, and induced cancer cell apoptosis. Lycoris alkaloid, a natural alkaloid obtained from the bulbs of Lycoris radiata (*Lycoris radiata*), was found to exert anti-tumor effects on H22 tumor-bearing mice in vivo according to Xin et al. [50] in 2020. This study demonstrated that lycorine can effectively suppress HepG-2 cell proliferation and significantly induce cell death. Borneol, an alkaloid compound naturally present in the plant borneol (*Borneolum Syntheticum*), is a commonly used component in Chinese herbal medicine. Research conducted by Li et al. [51] in 2021 on nanocarriers modified with borneol revealed that borneol can improve therapeutic efficacy, reduce toxicity, inhibit tumor metastasis, and reverse multidrug resistance. Moreover, berberine, an isoquinoline alkaloid isolated from Coptis chinensis (*Coptis chinensis*), was also widely explored as a potential natural anticancer therapeutic agent [52]. Achi et al. [53] confirmed in 2022 that berberine could enhance the anti-lung cancer efficacy by regulating several signaling pathways associated with cancer cell viability, proliferation, migration, invasion, and metastasis, as summarized in Table 3.

### 2.4. Polysaccharide

Angelica sinensis polysaccharides, one of the main active ingredients of Angelica sinensis (*Angelica sinensis*), have demonstrated considerable anti-tumor efficacy. As early as 2003, Shang et al. [60] confirmed that these polysaccharides exhibit anti-tumor effects in experimental tumor models in vivo and can inhibit liver cancer cell invasion and metastasis in vitro. Similarly, astragalus polysaccharides, a natural active ingredient extracted from the traditional Chinese medicine Huangshi (*Astragalus membranaceus*), has shown potential in cancer prevention. Li et al. [61] confirmed in 2019 that Huangshi polysaccharide can simulate macrophages to release NO and TNF-α to effectively prevent the growth of cancer cells, as summarized in Table 4.

In summary, the natural ingredients derived from Chinese herbal medicine exhibit multiple anti-tumor activities and mechanisms. They may exert multiple effects such as the regulation of tumor gene expression, immune system enhancement, and the induction of tumor cell differentiation. Consequently, these findings suggest substantial potential for the development of anti-tumor drugs based on these natural ingredients. Therefore, Chinese herbal medicine represents a valuable reservoir for anti-cancer drug discovery and development.

### 2.5. The Important Role of Cytochrome P450 in Traditional Chinese Medicine Active Substance Tumor Treatment

Cytochrome P450 is a large class of constitutive and inducible hemes containing enzymes that play a crucial role in human physiology and participate in drug and xenobiotic metabolism, as well as the biosynthesis of endogenous molecules, and are expressed throughout the human body [63]. Many P450 substrates are carcinogenic, while others are anticancer drugs. Therefore, P450 has various potential important roles in tumor biology [64].

Many natural compounds play a key role in anti-tumor therapy with cytochrome P450 [65]. Active substances in traditional Chinese Medicine, for example, genistein, may affect the expression level of cytochrome P450 involved in the biological transformation of exogenous substances and drug metabolism enzymes [66]. In human adrenal NCI-H295R cells, a certain concentration and dose of curcumin can effectively reduce the activity of cytochrome P450 enzyme, indicating that curcumin may inhibit steroid metabolism [67]. The key targets of Formonetin against colorectal cancer are cytochrome P450 3A4, tumor necrosis factor and cytochrome P450 1A1, and the molecular mechanism of Formonetin in the treatment of colorectal cancer may be related to the inhibition of cell proliferation and regulation of tumor-related metabolic pathways [68]. According to research, Rhein has obvious inhibitory effect on cytochrome P450 enzyme, which not only has strong antibacterial effect but may also play the role of biochemical regulator in cancer chemotherapy. Finally, the researchers also proposed that drug interactions, especially those mediated by cytochrome P450, can lead to the increased or decreased efficacy of combination therapy [69]. In addition, oridonin induces gene and protein expression as well as the enzyme activity of cytochrome P450 in human liver cancer cells, which may provide at least some basis for potential drug–drug interactions [70].

In addition to the positive effects of traditional Chinese medicine active substances on the regulation of cytochrome P450, some also have harmful effects. For example, Triptolide and its derivatives are considered to have good prospects for drug development. However, due to various organ toxicity, especially liver toxicity, the clinical application of Triptolide A is limited. Among them, Triptolide A-induced liver toxicity involves the regulation of cytochrome P450 enzymes [71].

In summary, cytochrome P450 acts on different molecular scaffolds, providing a unique and powerful enzyme toolbox that is crucial for the later functionalization of bioactive compounds for the production of new drugs.

## 3. The Synergistic Anti-Tumor Effect of Natural Components in Chinese Herbal Medicine Combined with Chemotherapy Drugs

Currently, numerous natural compounds derived from traditional Chinese medicine have been employed as adjunctive or alternative therapies for tumors. These are utilized to either enhance the synergy of chemotherapy drugs or mitigate their side effects [72]. Therefore, the components of Chinese herbal medicine that could be combined with chemotherapy treatment are identified and categorized based on their diverse synergistic anti-tumor mechanisms, which will be further discussed in the following sections.

### 3.1. Combination Strategies and Mechanisms of Synergistic Induction of Tumor Cell Apoptosis

Apoptosis is a programmed cell death pathway that has been extensively studied in various inflammatory disease and tumor models [73]. The induction of tumor cell apoptosis is one of the main mechanisms for both traditional chemotherapy drugs and extracts from Chinese herbal medicine with anti-tumor effects [74]. Numerous studies have shown that the combination therapy has significant synergistic anti-tumor effects, as listed in Table 5. Chio et al. [75] combined honokiol extracted from lignosomal compounds with the chemotherapy drug temozolomide and found that this combination can activate caspase-3 in U87-MG cells, thereby blocking the tumor cell growth cycle and ultimately inducing tumor cell apoptosis. This process synergistically enhances the anti-tumor effect of the chemotherapy drug temozolomide. In a separate study carried out in 2019, Bu et al. [76] found that Rhein combined with pemetrexed exhibited a significant anti-tumor effect. The study confirmed that Rhein regulates the PI3K-AKT-mTOR pathway and Bcl-2 protein family expression in lung cancer cells, thus enhancing pemetrexed’s anti-tumor activity by affecting cell apoptosis, as summarized in Table 5.

### 3.2. Combination Strategies and Mechanisms for Reducing Drug Resistance in Tumor Cells

Resistance to conventional chemotherapy is one of the key reasons for tumor treatment failure. Various potential mechanisms contribute to the development of drug resistance in tumor cells, including tumor heterogeneity, certain cellular level changes, genetic factors, etc. [90]. Therefore, understanding these mechanisms and their response strategies is crucial for inhibiting tumor cell resistance and enhancing anti-tumor drug sensitivity, which has significant effects on tumor treatment outcomes. Numerous studies have confirmed that combining natural anti-tumor ingredients extracted from Chinese herbal medicine with chemotherapy drugs can effectively reverse tumor multidrug resistance, as summarized in Table 5.

Abdallah et al. [77] investigated the efficacy of the natural flavonoid compound icariin combined with doxorubicin in the treatment of tumors in 2015 and found that icariin is able to increase the sensitivity of tumor cells to the chemotherapy drug doxorubicin, thereby reversing the drug resistance of tumor cells to doxorubicin. Then, Zhang et al. [78] found that genistein combined with the chemotherapy drug tamoxifen could reduce the de novo resistance of tamoxifen, thereby showing a better anti-tumor effect. Similarly, Lin et al. [79] studied the combination of natural isoliquiritigenin from Chinese herbal medicine and chemotherapy drug doxorubicin to treat uterine sarcoma cells. The results of their study confirmed that low doses of isoliquiritigenin can increase the sensitivity of tumor cells, reverse their resistance to chemotherapy drugs, and ultimately reduce the survival rate of tumor cells. In 2017, Zhou et al. [80] studied the natural anti-tumor component of Chinese herbal medicine, borneol, in combination with the chemotherapy drug paclitaxel to overcome multidrug resistance. They found that borneol can effectively increase the sensitivity of ovarian cancer cells to paclitaxel and reverse the defect of multidrug resistance, as summarized in Table 5.

### 3.3. Combination Strategies and Mechanisms for Synergistic Enhancement of Chemotherapy Drug Efficacy

As early as 2003, Zhang et al. [81] found that silymarin can enhance the cytotoxicity of doxorubicin in P-gp-positive cells, indicating a synergistic effect on enhancing cancer chemotherapy. In 2015, Yang et al. [82] used glycyrrhetinic acid as a carrier and combined it with the chemotherapy drug paclitaxel to form paclitaxel glycyrrhetinic acid micelles that can be used orally. The study found a significant increase in the oral absorption of paclitaxel from glycyrrhetinic acid micelles loaded with paclitaxel. Further research demonstrated that glycyrrhetinic acid increased the absorption of paclitaxel in the jejunum and colon, thus synergistically enhancing the anti-tumor effect of chemotherapy drugs. It could thus become a promising carrier for oral chemotherapy drugs. Subsequently, Liu et al. [83] confirmed in 2015 that formononetin may reverse the epithelial mesenchymal transition induced by chemotherapy drugs in tumor cells, thereby increasing the anti-tumor effect of chemotherapy drug doxorubicin.

In recent years, Hong et al. [84] combined ginsenoside Rg3 extracted from ginseng with the chemotherapy drug paclitaxel in 2019 to form a new multifunctional liposome system for the treatment of gastric cancer. Research confirmed that ginsenoside Rg3 acts as a chemotherapy adjuvant and membrane stabilizer, significantly enhancing the chemotherapy effect. In addition, Li et al. [85] confirmed in 2020 that a combination of lycorine extracted from lycoris bulbs and HA14-1 enhanced the therapeutic effect on gastric cancer and significantly inhibits tumor growth. These results indicated that the combination of anti-tumor components in Chinese herbal medicine and chemotherapy drugs has good application prospects in tumor treatment, which can effectively improve the treatment effect and safety, as summarized in Table 5.

### 3.4. Combination Strategies and Mechanisms for Reducing Adverse Reactions to Chemotherapy

Chemotherapy drugs are highly toxic, and platinum-based cancer chemotherapy drugs such as cisplatin and oxaliplatin are commonly used to treat various types of cancer [91]. However, their use is limited by side effects, especially adverse reactions such as vomiting, anorexia, and muscle atrophy, which are unbearable [91]. Therefore, strategies to reduce the toxicity of chemotherapy drugs deserve equal attention. In 2020, Wu et al. [86] studied the renal protective effect of puerarin on acute renal injury induced by chemotherapy drug cisplatin by upregulating the expression of miR-31 and inhibiting the Numb/Notch1 signaling pathway. In addition, Khan et al. [87] designed a pH-sensitive calcium carbonate drug co delivery system for the combination of oleanolic acid and chemotherapy drug cisplatin in 2022. The study found that oleanolic acid significantly reduced the nephrotoxicity induced by the chemotherapy drug cisplatin, thereby reducing the adverse effects of chemotherapy drugs, as summarized in Table 5.

### 3.5. Combination Strategies and Mechanisms as Chemotherapy Immunoadjuvant

According to investigations, certain Chinese herbal extracts can act as immune modulators for the immune system and have significant therapeutic effects in combination with chemotherapy drugs in the treatment of tumors. Wang et al. [88] collaborated with the chemotherapy drug doxorubicin to treat tumors in 2020, and found that Angelica polysaccharides not only serve as a carrier for targeted delivery of drugs to tumor tissue, but also have an effect on improving the tumor microenvironment and enhancing immune function. As a chemotherapy adjuvant, they have synergistic anti-tumor effects with chemotherapy drugs. Poria cocos polysaccharide is a traditional Chinese herbal medicine with multiple biological activities and prebiotic effects, the study by Lin Yin et al. [89] confirms that it can alleviate the cytotoxicity of 5-fluorouracil by reducing the expression of pro-inflammatory cytokines, increasing anti-inflammatory cytokines, and significantly improving the intestinal barrier by enhancing tight junction proteins and related adhesion molecules. Therefore, Poria cocos polysaccharide can improve the therapeutic effect of 5-fluorouracil by restoring the intestinal microenvironment and the homeostasis of gut symbiotic microbiota, as summarized in Table 5.

### 3.6. Combined Anti-Tumor Therapy of Traditional Chinese Medicine Active Ingredients and Chemotherapy Drugs Based on Nano-Delivery System

Although the active ingredients of traditional Chinese medicine exhibit good anti-tumor ability, their clinical application is still limited. The solubility and tumor targeting ability of most active ingredients are poor, leading to low bioavailability and reduced efficiency in delivering tumor targeting in vivo, posing several challenges [92]. Recently, many researchers have attempted to enhance the medicinal value of traditional Chinese medicine in treating and preventing diseases through delivery systems based on nanoparticles [93]. Compared with natural compounds, the combination of anti-tumor active ingredients of traditional Chinese medicine and chemotherapy drugs in the treatment of tumors based on nano delivery systems shows higher bioavailability, enhanced tissue targeting, higher in vivo activity, and higher stability of many active ingredients in this system, which indicates a good future development trend [94].

The specific nanocarriers include polymeric micelles, solid lipid nanoparticles, liposomes, polymeric nanoparticles, and nanogels. For example, the reversal of drug resistance in ovarian cancer cells with the combination therapy of borneol and paclitaxel mentioned above was mediated by PEG-PAMAM nanoparticles [80]. In the study of the combination of oleanolic acid and cisplatin for the treatment of tumors based on the lipid-coated cisplatin/oleanolic acid calcium carbonate nanoparticle delivery system, the investigators clearly indicated that a significantly long cycle time and constant therapeutic concentration of both drugs can be achieved through this drug delivery system [87]. A nano drug delivery system based on angelica sinensis polysaccharide for the combination of chemotherapy and immunotherapy, this drug delivery system with specific tissue targeting and tumor microenvironment improvement will open up a new avenue for cancer combination therapy [88], as summarized in Table 6.

Overall, these nano delivery systems can serve as a starting point for the use of traditional Chinese medicine active ingredients in anti-tumor therapy in the future. Therefore, we believe that with the advancement of technology, researchers will pay attention to the development of new drug delivery systems for traditional Chinese medicine anti-tumor active ingredients.

## 4. Current Challenges and Perspectives

During clinical treatment, long-term treatment with chemotherapy drugs can be limited by serious side effects, ultimately leading to poor patient prognosis. In recent years, a large amount of basic research has been conducted on active substances in traditional Chinese medicine, and it has been found that they have pharmacological activities such as inhibiting cancer cell proliferation, migration, and invasion, and promoting cell apoptosis. The combination of chemotherapy drugs and active substances in traditional Chinese medicine has shown promising results in cancer treatment. From the perspectives of economy, safety, and effectiveness, the combination of chemotherapy drugs and traditional Chinese medicine active substances is a promising tumor treatment.

But most combination therapies are developed based on experience, and there are few experimental studies aimed at thoroughly exploring different drug combinations using appropriate analytical methods. In particular, the combination of anti-tumor compounds in traditional Chinese medicine with chemotherapy drugs needs to consider the stability of the combination and the detailed mechanism of multi-drug anti-tumor effects, which is an urgent issue. Of course, drug combinations may also produce antagonistic effects, where the combined effect may not be as effective as a single drug, for example, one drug counteracts the action of another drug [95]. Therefore, controlling the dosage and time sequence of each drug combination requires precise control of the administration form to obtain the ideal drug combination.

In recent years, researchers have adopted nanocarriers to load active substances in traditional Chinese medicine, which can effectively solve these problems [96]. However, there are currently few reports on the use of multi-drug nano delivery for cancer treatment in clinical settings, and there are still some challenges that hinder treatment efficacy and clinical translation. In clinical use, it is necessary to consider the safety, allergic reactions, drug interactions, mechanisms of action, and biosafety of nanomaterials. Only when these issues are fully addressed can this combination therapy be widely applied in clinical practice. We believe that with further improvement by researchers, it has the potential to shine in clinical settings and bring good news to cancer patients.

## 5. Summary

In summary, compounds extracted from traditional Chinese medicine combined with chemotherapy drugs can prolong the survival period of tumor patients, improve long-term survival rate, and achieve therapeutic effects that cannot be achieved by simple chemotherapy. This demonstrates the important role of traditional Chinese medicine in the application of chemotherapy. However, it is worth noting that although the combination of extracts from Chinese herbal medicine and chemotherapy drugs has certain benefits in treating tumors, improper use can also lead to local and even systemic toxic reactions in patients, as well as damage to organs such as the heart, liver, and kidneys. Therefore, patients must comprehensively consider their own situation when choosing traditional Chinese medicine, and should not blindly accumulate and use it, nor should they solely rely on traditional Chinese medicine treatment and forego other conventional treatments.

Chemotherapy and traditional Chinese herbal medicine therapy each have certain advantages and disadvantages in tumor therapy. Although chemotherapy is currently the conventional treatment for tumors, it has significant side effects. Conversely, although Chinese herbal medicine has minimal side effects, it is difficult to extract compounds and its therapeutic effects on tumors can be weaker. Therefore, combining the advantages of the two treatment methods and overcoming each other’s shortcomings is the fundamental goal of combining traditional Chinese and Western medicine in treating tumors. This review summarizes and analyzes the anti-tumor strategies and mechanisms of traditional Chinese medicine combined with chemotherapy and finds that this combination therapy significantly prolongs the overall survival period of patients. However, current research results cannot clearly explain the advantageous population and mechanism of traditional Chinese medicine combined with anti-tumor therapy. More evidence-based experimental research is still needed to provide a basis for developing better cancer treatment combination strategies.

## Figures and Tables

**Table 1:** Examples of polyphenolic compounds with anti-tumor effects in Chinese herbal medicine.

Natural Anti-Tumor Ingredients of Chinese Herbal Medicine	Molecular Structure	Source	Part of Plant	Target and IC50	Cancer	Refs.
Genistein	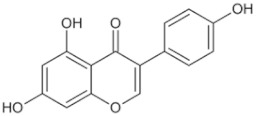	Legume	_	MCF-7 (IC50 = 9.4 μM), T47D ER + (IC507 = μM)	Prostatic cancer	[14,25]
Silymarin	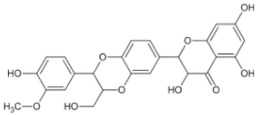	Milk thistle fruit	Seeds, fruits	HepG2 (IC50 = 58.46 μM)	Liver cancer	[15,26]
Curcumin	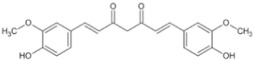	Turmeric	Roots	AGS (IC50 = 2 1.9 ± 0.1 μM), HT 29 (IC50 = 40.7 ± 0.5 μM)	Gastric cancer	[23,27]
Honokiol	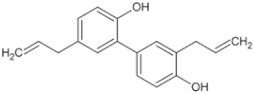	Traditional Chinese Medicine Houpu	Bark	MDA-MB-231 (IC50 = 16.99 ± 1.28 μM), MDA-MB-468 (IC50 = 15.94 ± 2.35 μM), MDA-MB-453 (IC50 = 20.11 ± 3.13 μM)	Prostatic cancer, bladder cancer	[24,28]
Tanshinone ⅡA	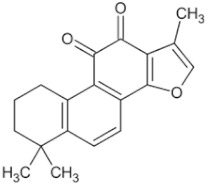	Salvia miltiorrhiza	Roots, rhizomes	A549 (IC50 = 145.3 μM)	Lung cancer, human nasopharyngeal carcinoma	[21,29]
Isoliquiritigenin	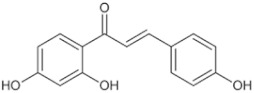	Derived from licorice and carnation	Roots, rhizomes	RBC (IC50 = 2.0 μM)	Multiple cancers	[16,30]
Formononetin	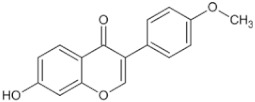	Astragalus membranaceus, Sophora flavescens, licorice, kudzu root, and other leguminous plants	Roots	SW480 (IC50 = 4.31 μM)	Colorectal cancer, cervical carcinoma	[18,31]
Puerarin	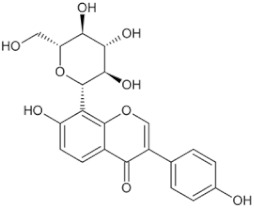	Pueraria lobata	Roots	BUC T24 (IC50 = 218 μM)	Bladder cancer	[19,32]
Rhein	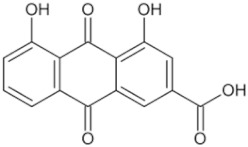	Rheum palmatum	Roots, rhizomes	MGC803 (IC50 = 94.26 μM)	Gastric cancer, liver cancer	[22,33]
Icariin	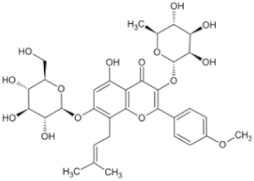	Epimedium plant	Rhizomes	KYSE70 (IC50 = 40 μM)	Esophageal cancer, ovarian cancer	[20,34]

**Table 2:** Examples of terpenoids with anti-tumor effects in Chinese herbal medicine.

Natural Anti-Tumor Ingredients of Chinese Herbal Medicine	Molecular Structure	Source	Part of Plant	Target and IC50	Cancer	Refs.
Ginsenoside Rg3	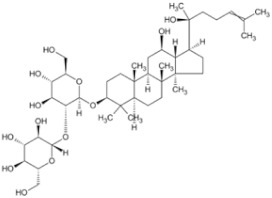	Ginseng	Roots	_	Breast tumors	[35]
Oridonin	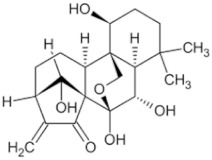	Chinese medicine Rabdosia rubescens	_	KYSE70 (IC50 = 8.4 μM)	Esophageal cancer	[36,41]
Triptolide	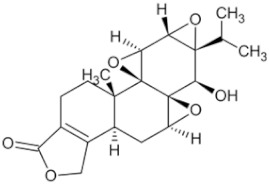	Tripterygium wilfordii plant	Roots	Capan-1 (IC50 = 0.01 μM)	Pancreatic cancer, breast cancer	[37,42,43]
Ganoderma triterpenes	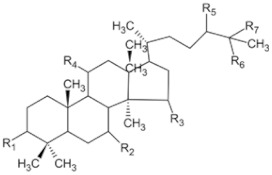	Ganoderma lucidum	The surface part of gills	_	Lung cancer	[38,44]
Oleanolic acid	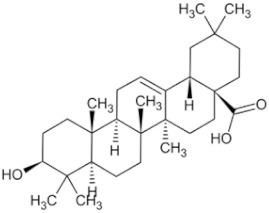	In plants, such as the leaves of the Oleaceae plant Qidunguo	Roots	HepG2 (IC50 = 31.94 ± 1.03 μM)	Liver cancer	[39,45,46]
Glycyrrhizic acid	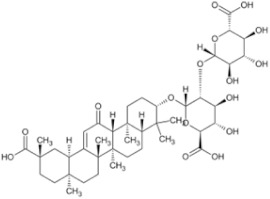	Licorice	Roots, rhizomes, leaf	SW480 (IC50 = 210 μM)	Colorectal cancer, melanoma	[40,47]

**Table 3:** Examples of alkaloid compounds with anti-tumor effects in Chinese herbal medicine.

Natural Anti-Tumor Ingredients of Chinese Herbal Medicine	Molecular Structure	Source	Part of Plant	Target and IC50	Cancer	Refs.
Camptothecin	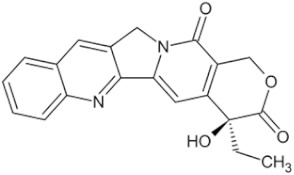	Camptotheca acuminata fruit	Seed, root bark	HCC1419 (IC50 = 0.067 μM)	Multiple cancers	[48,54,55]
Chelidonine	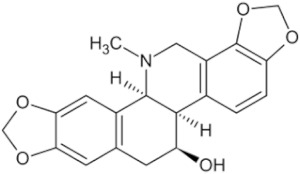	Baiqu Cabbage	Leaf	A-375 (IC50 = 3 μM), U2OS (IC50 = 34.51 ± 9.47 μM)	Liver cancer, lung cancer, melanoma	[49,56,57]
Lycorine hydrochloride	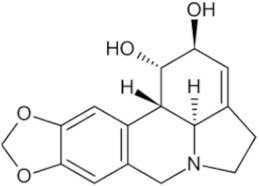	Lycoris bulb	_	Hey1B (IC50 = 1.2 μM)	Endometrial cancer, Liver cancer	[50,58]
Borneol	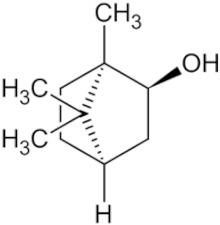	Borneol fragrance	_	_	Multiple cancers	[51]
Berberine	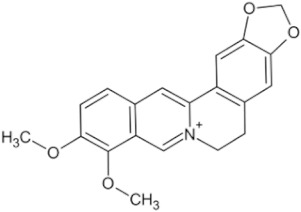	Coptis chinensis	Roots, rhizomes, stem, bark	LoVo (IC50 = 1.25–160 μM)	Colorectal cancer, Lung cancer	[52,59]

**Table 4:** Examples of polysaccharides with anti-tumor effects in Chinese herbal medicine.

Natural Anti-Tumor Ingredients of Chinese Herbal Medicine	Molecular Structure	Source	Part of Plant	Target and IC50	Cancer	Refs.
Angelica polysaccharide	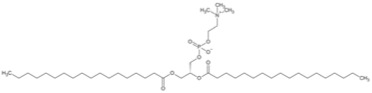	Angelica	Roots	_	Liver cancer	[60]
Astragalus polysaccharides	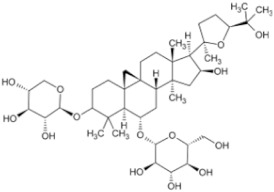	Mongolian Milkvetch Root	Roots	HepG2 (IC50 = 4.2 μM)	Liver cancer, breast cancer	[61,62]

**Table 5:** The synergistic anti-tumor effect of natural anti-tumor components of Chinese herbal medicine combined with chemotherapy drugs.

Cooperative Mechanism	Natural Active Ingredients of Chinese Herbal Medicine	Combined Chemotherapy Drugs	Cancer	Molecular Mechanism	Refs.
Synergistic induction of tumor cell apoptosis	Honokiol	Temozolomide	Glioma	The combined treatment of human U87-MG cells with magnolol and temozolomide can induce greater caspase-3 activation, DNA fragmentation, cell apoptosis, and cell cycle arrest in the G1 phase.	[75]
	Rhein	Pemetrexeddisodium for injection	Lung cancer	Rhein enhances the anti-tumor activity of paclitaxel by regulating the PI3K-AKT-mTOR pathway and Bcl-2 protein family in A549 cells, affecting autophagy and apoptosis.	[76]
Reduce drug resistance of tumor cells	Icariin	Doxorubicin	Multiple cancers	Rhein enhances the anti-tumor activity of paclitaxel by regulating the PI3K-AKT-mTOR pathway and Bcl-2 protein family in A549 cells, affecting autophagy and apoptosis.	[77]
	Genistein	Tamoxifen	Breast cancer	The downregulation of unfolded protein response and autophagy-related genes and genes linked to immunosuppression and upregulation of cytotoxic T-cell marker CD8a in the tumors of the lifetime genistein group, compared with controls groups.	[78]
	Isoliquiritigenin	Doxorubicin	Uterine sarcoma	Isoliquiritigenin inhibits cell growth by inducing apoptosis and autophagy via inhibition of m-TOR signaling.	[79]
	Borneol	Paclitaxel	Ovarian cancer	Based on the synergistic effect of paclitaxel and borneol combination on multidrug resistance reversal by impairing drug efflux resulted from the over-expressed P-gp function.	[80]
Synergistic enhancement of chemotherapy drug efficacy	Silymarin	Doxorubicin	Colorectal cancer	Biochanin A and silymarin can inhibit P-gp-mediated efflux in Caco-2 cells.	[81]
	Glycyrrhizic acid	Paclitaxel	Colorectal cancer	Glycyrrhizic acid can increase the solubility of hydrophobic drugs and their permeability through cell membranes, as it can increase membrane permeability and reduce membrane elasticity.	[82]
	Formononetin	Doxorubicin	Glioma	Formononetin sensitizes glioma cells to doxorubicin through preventing epithelial–mesenchymal transition via the inhibition of histone deacetylase 5.	[83]
	Ginsenoside Rg3	Paclitaxel	Gastric cancer	The combination therapy using these targeted liposomes significantly suppressed gastric cancer tumor growth.	[84]
	Lycorine	HA14-1 (inhibitor of BCL2)	Gastric cancer	Lycorine hydrochloride reduced the protein stability of MCL1 by up-regulating ubiquitin E3 ligase FBXW7, arrested cell cycle at S phase and triggered the apoptosis of gastric cancer cells.	[85]
Reduce adverse reactions to chemotherapy	Puerarin	Cisplatin	Acute renal injury	Cisplatin increased the expression levels of apoptotic proteins and affected the Numb/Notch1 signaling pathway, which is downstream of miR-31.	[86]
	Oleanolic acid	Cisplatin	Liver cancer	Oleanolic acid greatly reduced cisplatin induced nephrotoxicity in the formulation.	[87]
As an immunoadjuvant for chemotherapy	Angelica polysaccharide	Doxorubicin	Multiple cancers	The treatment of released AP moiety increased the expression of IL-2, while that of IL-10 was decreased, showing potential in restoring Th1/Th2 immune balance in tumor microenvironment.	[88]
	Poria cocos polysaccharides	5-Fluorouracil	Multiple cancers	Poria cocos polysaccharide alleviates the cytotoxic effects of 5-fluorouracil by reducing the expression of pro-inflammatory cytokines, increasing anti-inflammatory cytokines, and significantly improves the intestinal barrier by enhancing tight junction proteins and related adhesion molecules.	[89]

**Table 6:** Summary of combined anti-tumor effects of traditional Chinese medicine active ingredients and chemotherapy drugs based on nano delivery systems.

Nano-Carriers	Formulations	Traditional Chinese Medicine Active Ingredients	Combined Chemotherapy Drugs	Cancer	Refs.
Polymeric nanoparticles	PEG-PAMAM nanoparticle	Borneol	Paclitaxel	Ovarian cancer cells	[80]
Solid lipid nanoparticles	Lipid-coated cisplatin/oleanolic acid calcium carbonate nanoparticles	Oleanolic	Cisplatin	Liver cancer	[87]
Polymeric nanoparticles	Enzyme-sensitive tumor-targeting nano drug delivery system	Angelica sinensis polysaccharide	Doxorubicin	Multiple cancers	[88]

## Data Availability

Data sharing is not applicable.
